# Muscular Strength Spurts in Adolescent Male Basketball Players: The INEX Study

**DOI:** 10.3390/ijerph18020776

**Published:** 2021-01-18

**Authors:** Eduardo Guimarães, José A. R. Maia, Mark Williams, Filipa Sousa, Eduardo Santos, Fernando Tavares, Manuel A. Janeira, Adam D. G. Baxter-Jones

**Affiliations:** 1 Centre of Research, Education, Innovation and Intervention in Sport (CIFI2D), Faculty of Sport, University of Porto, 4200-450 Porto, Portugal; jmaia@fade.up.pt (J.A.R.M.); filipas@fade.up.pt (F.S.); esantos@fade.up.pt (E.S.); ftavares@fade.up.pt (F.T.); janeira@fade.up.pt (M.A.J.); 2 Department of Health and Kinesiology, College of Health, University of Utah, Salt Lake City, UT 84108, USA; mark.williams@health.utah.edu; 3 College of Kinesiology, University of Saskatchewan, Saskatoon, SK S7N 5B2, Canada; baxter.jones@usask.ca

**Keywords:** peak physical performance, age-at-peak height velocity, growth rate, youth athletes

## Abstract

Although successful performance in basketball requires high levels of muscular strength during adolescence, its development is confounded by the effects of normal growth. We examine the timing, intensity and sequence of muscular strength according to biological age (years from peak height velocity (PHV)) and hypothesize that young basketball players attain their peak muscular strength spurts around PHV. A total of 160 adolescent male basketballers, aged 11–15 years, were followed bi-annually over 3 consecutive years. The years from attainment of PHV and peak weight velocity (PWV) were estimated and five muscular strength measures (sit-ups, handgrip, seated medicine ball throw, squat jump and countermovement jump) were aligned to years from PHV in 3-month intervals. Strength velocities were estimated using a non-smooth mathematical model. The mean ages at-PHV and at-PWV were 13.90 ± 1.40 years and 13.90 ± 1.79 years, respectively. Maximal velocity in sit-ups was attained 6 months prior to attainment of PHV (intensity = 10.69 repetitions·year^−1^), whereas maximal velocity in squat jump occurred 6 months after-PHV (intensity = 3.93 cm·year^−1^). Handgrip strength, seated medicine ball throw and countermovement jump maximal velocity peaked at-PHV (intensity = 8.47 kgf·year^−1^, intensity = 0.75 m·year^−1^, intensity = 5.59 cm·year^−1^, respectively). In general, maximal velocity spurts did not differ in their timing, with the velocities reaching a peak concurrent with PHV and PWV or within 6 months of its attainment. Basketball coaches, as well as strength and conditioning trainers, should consider individual differences in strength development and be aware of rapid periods of growth in stature when planning and designing muscular strength training regimes.

## 1. Introduction

The adolescent growth spurt, in height, an index of biological maturation, varies substantially in timing, intensity and duration between individuals [1]. Within the same chronological age group, boys advanced in biological maturation tend to be physically fitter and perform better on muscular strength tasks [2]. This statement is true for young athletes [3], including adolescent basketballers [4]. Thus, in order to control for the variability in the adolescent growth spurt (peak height velocity (PHV)), there is a need to align muscular strength data with biological maturation/age (years-from-PHV) to accurately interpret and understand muscular strength development in relation to maturity [5].

Basketball is a highly dynamic and intermittent sport requiring remarkable physical attributes to excel in training and competition [6]. It is characterized by its multidirectional movements and intense activities like jumping, sprinting, shuffling and changes of direction, as well as by technical skills such as catching, dribbling, shooting and passing [7]. These specific characteristics require high levels of muscular strength. It is well-acknowledged that developing adequate levels of muscular strength should be a priority for adolescent athletes in their commitment towards excellence [8]. Furthermore, it is reported that improved strength reduces the risk of injury and plays an important role in learning and enhancing motor aptitudes and game-related skills [9]. However, care must be taken when interpreting data on muscular strength in young athletes because it is confounded by inter-individual differences in biological maturation [2].

Published reports on developmental spurts in muscular strength mostly focus on non-athletic populations and vary in timing and intensity when aligned to years-from-PHV. For example, in standing long jump (a marker of explosive leg strength) the peak spurt occurred 12 months before-PHV (intensity = 22.3 cm·year^−1^) in a group of boys from Portugal [10], 6 months before-PHV (intensity = 28.5 cm·year^−1^) in boys from Brazil [11], was concurrent with PHV (intensity = 15.0 cm·year^−1^) in boys from Canada [12], but in boys from Spain, a plateau was reported between PHV and 12 months post-PHV (intensity = 21.0–22.0 cm·year^−1^) [13]. A similar level of variation in timing and intensity is reported in other measures of muscular strength such as sit-ups, handgrip and arm pull [2,10,11,12,13,14]. However, to the best of our knowledge, only one published report exists using adolescent athletes. Philippaerts et al. [15] identified peak spurts in a variety of motor performance traits in soccer players, while components of muscular strength (i.e., sit-ups, bent arm hang, standing long jump, and vertical jump) peaked around the same time as PHV. We contend that the systematic investigation of muscular strength peak spurts, as well as their sequence, has not been identified in adolescent athletes. Therefore, in this paper, we expand this knowledge to a team sport by identifying the timing, intensity and sequence of peak spurts of distinct muscular strength components in young basketball players. Since coaches prioritize their training regimens in terms of muscular strength development, linking these spurts to skill development and game performance is of upmost importance [8]. Furthermore, these links may be relevant to connect strength development to known periods of training sensitivity. Such periods may offer critical windows of opportunity to enhance responses to training and competition [16]. It has been hypothesized that the use of inadequate training loads during these windows of opportunity may limit the expression of athletic potential [17]. Notwithstanding the relevance of this suggestion, no empirical evidence exists on the topic. The aims of the present study are to investigate the timing, intensity and sequence of development of muscular strength spurts in male basketballers aligned by biological age (years-from-PHV). We hypothesize, based on previous findings [15], that young basketball players attain peak muscular strength spurts around PHV.

## 2. Materials and Methods

### 2.1. Participants

The data presented in the current paper comes from the *In Search of Excellence—a Mixed-longitudinal Study in Young Athletes* (INEX) study. The study involves a 3-year mixed-longitudinal approach consisting of five age-cohorts from five team sports (https://www.inex-cifi2d.pt). The purpose of the INEX study was to investigate the interactions among individual characteristics and environmental factors affecting growth, physical and specific-skills performance, game proficiency and psychological development.

In the present study, data were collected bi-annually over 3 consecutive years from a sample of 293 male adolescent basketballers. Altogether, 160 players fulfilled the condition of having complete data on 5 to 6 time points for anthropometry and muscular strength measures. In cohort 1 (*n* = 31), we followed players consecutively from 11 to 13.5 years, in cohort 2 (*n* = 40) from 12 to 14.5 years, in cohort 3 (*n* = 32) from 13 to 15.5 years, in cohort 4 (*n* = 33) from 14 to 16.5 years, and in cohort 5 (*n* = 24) from 15 to 17.5 years. Participants were recruited from a population of 1256 adolescent male basketball players. All trained regularly 4.5 to 6.0 h·week^−1^ with 20 of the 25 clubs in the Porto Basketball Association. Participants were randomly selected by coaches and club coordinators to take part in data collection. Baseline measurements were initiated in June of 2017 and repeated through to December of 2019. All assessments occurred during the same time-periods (June and December) within a time window of 15–20 days. Written informed consent was obtained from parents or legal guardians, as well as players’ individual assents. The study was approved by the Ethics Committee of the lead institution (CEFADE 13.2017). The Porto Basketball Association gave formal permission for data collection.

### 2.2. Anthropometry

Height (cm) was measured without shoes and with the participant’s head positioned in the Frankfurt plane using a Harpenden stadiometer (Holtain Ltd., Crymych, UK) with a precision of 0.1 cm. Weight (kg) was measured using a bio-impedance scale (Tanita ^®^ BC-418MA, Tanita Corp., Tokyo, Japan) with a precision of 100 g. Measurements were assessed following the International Working Group on Kinanthropometry protocols [18].

### 2.3. Muscular Strength

Muscular strength was assessed using standardized tests: (1) Abdominal muscular strength and endurance (sit-ups): the maximum number of sit-ups (repetitions (reps)) during 60 s were recorded [19]; (2) Static strength (handgrip): a maximal handgrip strength (kgf) was obtained using a hand-held dynamometer (Takei Digital Grip Strength Dynamometer Model T.K.K.5401, Takei Scientific Instruments Co., Ltd., Tokyo, Japan) [20]; (3) Upper body explosive strength (3 kg seated medicine ball throw): distance (m) of a straightforward throw while seated on the floor with legs fully stretched and back against the wall was recorded [21]; (4) Lower body explosive strength (squat jump and countermovement jump): two vertical jumps were performed as advocated by Bosco et al. [22] using a AMTI OR6-WP force platform (Advanced Mechanical Technology Inc., Watertown, MA, USA) operating at 2000 Hz; jumping height (cm) was obtained.

### 2.4. Data Quality Control

A four-step procedure was used: (1) anthropometric measurements were performed by experienced anthropometrists; (2) an in-field reliability study was performed using a random sample of three-to-five participants per day; (3) reliability estimates were computed and the technical error of measurement (TEM) for height was 0.2 cm and for weight was 0.1 kg, whereas the ANOVA-based intraclass correlations (R) values for the muscular strength tests ranged from 0.90 (sit-ups) to 0.99 (seated medicine ball throw); (4) data cleaning was undertook to control for errors in data entry and the presence of outliers.

### 2.5. Statistical Analysis

Maximum velocities for height, weight and muscular strength tests were fitted using a modified non-smoothed polynomial method. This method was initially proposed by Van’t Hof et al. [23] and subsequently used by Beunen et al. [2], Yagüe and De La Fuente [13], and Philippaerts et al. [15]. A mathematical generalization of this methodology was developed and used by our research group [10,11]. We developed mean velocity curves and defined these in terms of time (i.e., months before and after-PHV). Although measurements were taken every six-months, the method allows the estimation of individual velocities every three-months. We estimated growth velocities using the Peak Growth software developed by a mathematician and software programmer from the University of Porto. Graphical data were displayed using a cubic spline procedure, implemented in GraphPad Prism v.8 (GraphPad Software Inc., San Diego, CA, USA). A cubic spline employs interpolating cubic polynomials and uses information from neighboring points to obtain a degree of global smoothness. The cubic spline was chosen over other curve fitting procedures because it maintains the integrity of the data without transforming or modifying the underlying growth characteristics.

## 3. Results

**Figure 1:**
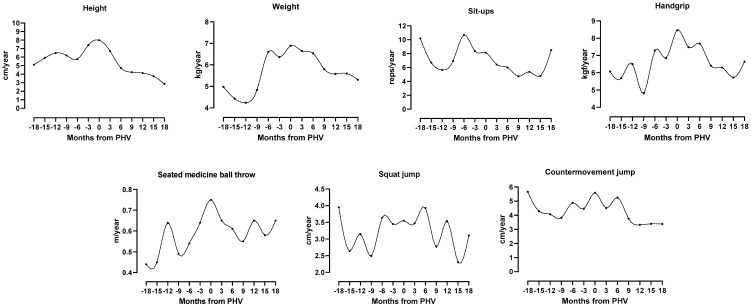
Mean velocity curves for height, weight and muscular strength components aligned by biological age (months from peak height velocity (PHV)); 0 = PHV.

The mean ages at-PHV and at-peak weight velocity (PWV) were 13.90 ± 1.40 years and 13.90 ± 1.79 years, respectively. Height velocity increased from 5.12 cm·year^−1^ at 18 months before-PHV to 7.99 cm·year^−1^ at-PHV. The velocity then rapidly decreased to 2.86 cm·year^−1^ at 18 months after-PHV. Weight velocity increased from 4.98 kg·year^−1^ at 18 months prior-PHV to 6.89 kg·year^−1^ at-PHV and thereafter dropped to 5.31 kg·year^−1^ at 18 months post-PHV. The timing and intensity of height, weight and muscular strength peak spurts are shown in Table 1 and graphically illustrated in Figure 1.

For sit-ups, the mean velocity decreased from 10.19 reps·year^−1^ at 18 months before-PHV to 5.65 reps·year^−1^ at 12 months before-PHV. Subsequently, there was an acceleration with a peak (10.69 reps·year^−1^) 6 months before-PHV and then dropped to 4.74 reps·year^−1^ at 9 months after-PHV. Thereafter, velocities slightly increased to 5.37 reps·year^−1^ at 12 months after-PHV, decreased to 4.79 reps·year^−1^ at 15 months after-PHV and increased again to 8.52 reps·year^−1^ at 18 months after-PHV.

For handgrip, the average velocity curve showed a fluctuation between 6.07 kgf·year^−1^ at 18 months before-PHV and 6.85 kgf·year^−1^ at 3 months before-PHV. Thereafter, the velocity increased attaining a peak (8.47 kgf·year^−1^) coincident with PHV. The velocity then declined to 5.72 kgf·year^−1^ at 15 months after-PHV but increased again to 6.64 kgf·year^−1^ at 18 months after-PHV.

For seated medicine ball throw, the velocity rose from 0.44 m·year^−1^ at 18 months before-PHV to 0.64 m·year^−1^ at 12 months before-PHV but declined right after to 0.49 m·year^−1^ at 9 months before-PHV. It then accelerated reaching a peak (0.75 m·year^−1^) coincident with PHV. Subsequently, the velocity decreased to 0.55 m·year^−1^ at 9 months after-PHV and fluctuated until 0.65 m·year^−1^ at 18 months after-PHV.

In squat jump, the velocity curve displayed a fluctuation between 18 and 3 months before-PHV, followed by a plateau until 3 months after-PHV. Thereafter, the velocity increased resulting in a maximum peak velocity of 3.93 cm·year^−1^ at 6 months after-PHV. Then, the curve fell to 2.77 cm·year^−1^ at 9 months after-PHV and fluctuated until 3.10 cm·year^−1^ at 18 months after-PHV.

For countermovement jump, the velocity curve decreased from 18 months before-PHV until 9 months before-PHV. It then accelerated reaching a peak (5.59 cm·year^−1^) coincident with PHV, followed by a decline to 3.32 cm·year^−1^ at 12 months after-PHV and a plateau thereafter until 18 months after-PHV.

## 4. Discussion

In this novel study, we report estimated age-at-PHV, age-at-PWV and the timing, intensity and sequence of muscular strength spurts in a sample of young male basketballers from Portugal. Due to the absence of published reports using biological age (age-at-PHV) to identify peak spurts in muscular strength in young basketballers, we limit comparisons to Philippaerts et al. [15], the only available study with adolescent athletes. When making comparisons and interpretations, discrepancies may arise and these may be due to the time lag in publication, differences in sampling, study duration, presumed differences across countries and the mathematical model used to estimate growth spurt parameters. For example, although we have a total sample of 160 participants, PHV was only identified in 159 players, whereas successful fitted data were only available for 33 out of 76 young soccer players [15]. Additionally, the sub-samples used to estimate peak spurts varied by muscular strength component. We identified a peak in handgrip in 117 out of 160 basketballers, whereas Philippaerts et al. [15] found a peak in standing long jump in 10 out of 33 soccer players.

The average age-at-PHV was 13.90 ± 1.40 years, which did not differ to the 13.8 ± 0.8 years estimated in a sample of soccer players from Belgium [15] but relatively earlier than the 14.2 ± 0.9 years found in basketball players from Czechoslovakia (also including 1 athletic runner) [24] and soccer players from Wales [25] and Denmark [26]. Our estimated age-at-PHV was comparable to non-athletic boys from Switzerland, 13.9 ± 0.8 years [27] but relatively earlier than boys from Belgium and Sweden, 14.2 ± 1.0 years [2] and 14.1 ± 1.1 [28], respectively. Additionally, the estimated PHV intensity for basketballers (7.99 cm·year^−1^) was relatively lower than those reported by Šprynarová [24] (10.1 cm·year^−1^), Philippaerts et al. [15] (9.7 cm·year^−1^), and Bell [25] (9.5 cm·year^−1^). The maximum height velocity in basketballers was relatively lower than in samples of non-athletes from the UK, Sweden, Belgium, and Switzerland at 10.3 cm·year^−1^ [29], 9.8 cm·year^−1^ [28], 9.2 cm·year^−1^ [2], and 9.0 cm·year^−1^ [27], respectively. It is possible that the uniqueness of each sample, the temporal trend, distinct methods used to estimate age-at-PHV and its intensity may be responsible for these differences. However, our results are consistent with the ranges reported by Malina et al. [3] for team sport athletes across Europe (age-at-PHV: 12.8 to 14.5 years; intensity: 9.3 to 10.1 cm·year^−1^).

The mean age-at-PWV for our basketballers was 13.90 ± 1.79 years. This value was relatively similar to the 13.8 ± 0.8 years estimated in soccer players from Belgium [15], but relatively earlier than the 14.5 ± 1.0 years found in basketballers from Czechoslovakia (also including 1 athletic runner) [24] and 14.3 years in soccer players from Wales [25]. Additionally, our estimated age-at-PWV was relatively earlier than those reported in non-athletic boys from Sweden [28], the UK [29] and Belgium [2], namely, 14.3 ± 1.1 years, 14.3 ± 0.1 years, and 14.6 ± 1.2 years, respectively. Furthermore, young basketballers’ estimated PWV intensity (6.89 kg·year^−1^) was relatively lower than those reported by Šprynarová [24] (10.1 kg·year^−1^), Bell [25] (9.1 kg·year^−1^) and Philippaerts et al. [15] (8.4 kg·year^−1^). Similar to the age-at-PWV, maximum weight velocity found in our basketballers was relatively lower than the 9.8 kg·year^−1^ reported in non-athletic boys from the UK [29], 9.1 kg·year^−1^ from Sweden [28] and 8.8 cm·year^−1^ from Belgium [2]. As with age-at-PHV, these results might be explained by sample specificities, the temporal gap between studies, as well as different mathematical techniques used to estimate age-at-PWV.

For the 60 s sit-ups, the peak was identified 6 months before-PHV (intensity = 10.69 reps·year^−1^), whereas in soccer players from Belgium, the peak for 30 s sit-ups was coincident with PHV with an intensity of 2.7 reps·year^−1^ [15]. Ellis et al. [12] identified a peak in 60 s sit-ups 12 months prior-PHV (intensity = 2.8 reps·year^−1^) in a sample of boys from Canada, whereas Yagüe and De La Fuente [13] found a peak in 30 s sit-ups 16 months prior to PHV (intensity = 5.4 reps·year^−1^) in boys from Spain, even though there was a second period of rapid development 4 months after-PHV (intensity = 4.6 reps·year^−1^). The differences in timing may be explained by, at least, five factors: (1) tests do not have the same time length (30 s versus 60 s), (2) samples vary in their size and comprise athletes and non-athletes, (3) the use of a different mathematical method to estimate age-at-PHV and corresponding spurts, (4) the time lag between studies, and (5) the country of origin. In any case, the most striking results concern spurt intensities. For example, the intensity of soccer players from Belgium was about half that of non-sporting youth from Spain in spite of their training schedule (3 h·week^−1^) plus a competitive game during the weekend. Apparently, training and competition did not have enough stimuli to optimize levels of abdominal muscle strength and endurance in young soccer players. On the other hand, the peak intensity in basketballers in Portugal was double that of their non-sporting peers in Canada. The coaches in Portugal emphasize developing a stronger core, that is frequently linked to correct posture, stability, balance, support of the lower back, and reduced injury risk. They also develop a solid base to improve other muscular strength components that are important for game actions like jumping, throwing, sprinting, shuffling or changes of direction [30].

The handgrip strength spurt coincided with PHV (intensity = 8.47 kgf·year^−1^). Comparable results were only available for non-athletic boys from Portugal and Brazil. Guimarães et al. [10] identified peaks in handgrip strength coincident and 12 months after-PHV (intensity = 6.1 and 6.1 kgf·year^−1^, respectively), whereas Silva et al. [11] found it 9 to 12 months also after-PHV (intensity = 8.4 and 8.2 kgf·year^−1^, respectively). Similar timings, after-PHV, were reported in other populations of adolescent males, although using different static strength markers [2,14]. In our study, however, handgrip strength reached a maximal velocity coincident with the height spurt, which contrasts with previous suggestions linking the spurt in static strength to the adolescent weight spurt [2]. Although we anticipate that sample specificities or distinct methodologies might be responsible for these minor differences, it is possible that in young athletes, regular and systematic training play an important role to static strength spurt timing. Body size matters in basketball to identify a player’s specific position and to explain individual or team success [31]. Thus, it is possible that height and weight positively contributed, in absolute terms, to handgrip performance [32]. Additionally, the continuous use of arms and hands during basketball training sessions as well during all games may explain the higher peak gain of the basketballers. Players are systematically engaged in the actions of catching, holding and throwing the ball, which are closely linked to several defensive (rebounds, steals, interceptions) and offensive (lay-ups, jump shots, dribble, passes, rebounds, fakes) ball handling skills [33].

The peak spurt in 3 kg seated medicine ball throw occurred coincident with PHV (intensity = 0.75 m·year^−1^), which contrasts with Yagüe and De La Fuente [13] report, that is, 4 months after-PHV (intensity = 1.38 m·year^−1^). Nonetheless, our results are consistent with reports from the Ghent Youth Soccer Project [15], the Leuven Growth Study of Belgian Boys [2], and the Saskatchewan Growth Study [12], in that peak spurt timing is coincident with PHV, in spite of the fact that bent arm hang and flexed arm hang were used to assess upper body muscular strength. In any case, results concerning peak intensity deserve clarification, since in basketballers the intensity was smaller than reported in non-athletic boys from Spain (less 0.63 m·year^−1^). It is possible that distinct test protocols may explain this difference, although in both studies a medicine ball with equal weight (3 kg) was used. Young basketballers had to throw it while seated on the floor with legs fully stretched and back against the wall, whereas in the study conducted in Spain it is not known which test protocol was used (seated, standing or overhead). It should be noted, however, that basketballers continued to develop their explosive arm strength after attaining peak spurt. In fact, the velocity curve reaches twice an intensity of 0.65 m·year^−1^ (12 and 18 months after-PHV), in contrast to the sudden drop in velocity (0.40 m·year^−1^ at 16 months after-PHV) observed in non-athletic boys from Spain. It is possible that this continued improvement reflects stimuli and neuromuscular adaptations promoted by repeated actions of shooting, passing, controlling and dribbling during basketball trainings and games. In addition, it may be that basketball coaches invest in strength and conditioning workouts that enhance synchronization and coordination of upper body segments, given its contribution to a more effective skill domain in ball throwing [34].

Peak spurts in squat and countermovement jump were attained 6 months after-PHV (intensity = 3.93 cm·year^−1^) and coincident with PHV (intensity = 5.59 cm·year^−1^), respectively. While we were unable to locate any study reporting these two types of jump, our results are in part comparable with data from athletes and non-athletic boys from Belgium. Using a vertical jump test in which participants were instructed to bend their knees and swing their arms before jumping as high as possible, that is, benefiting from the stretch-shortening cycle as basketballers in the countermovement jump, Philippaerts et al. [15] reported a peak coincident with PHV (intensity = 5.1 cm·year^−1^), whereas Beunen et al. [2] found it 6 months after-PHV (intensity = 4.8 cm·year^−1^). Furthermore, a growth spurt in lower body explosive strength was identified a year after-PHV by Yagüe and De La Fuente [13], but with the standing long jump. We contend that systematic and regular sport training impacts on explosive strength peak timing. However, the intensity of the peak in basketballers was higher than in soccer players. This finding may be linked to the amount of weekly time basketballers engaged in training and competition that involved a large number of jumps [35]. Moreover, it may suggest that coaches invest in plyometric workouts, recognizing its importance to develop, for example, players’ ability to perform uncontested lay-ups and jumps shots or to grab more rebounds during the game.

The pattern in muscular strength peaks was similar to that observed in young athletes from Belgium [15]. When aligned by years from PHV, the identified sequence showed that abdominal muscular strength and endurance peaks just before-PHV, static strength and upper body explosive strength peaks coincident with PHV, and lower body explosive strength peaks at or just after-PHV. This pattern suggests that muscular strength spurts are linked to increases in plasma testosterone that occur during the year of PHV [36], aligning with Grosser et al. [37] suggestions that youth male athletes pass through a sensitive period between 11/13 and 15/17 years of age (around the year of PHV) in which gains in muscular resistance and explosive strength can be maximized. According to Scott [38], the theory of critical periods states that the organization of a system is most easily modified during the time of most rapid development. This theory implies the presence of specific time periods (windows of opportunity) during which an individual may be highly sensitive to environmental influences and assumes that several changes underlying physical growth, biological maturation and development occur more quickly during this time [39]. Therefore, if precisely identified, sensitive periods might represent moments of maximal readiness [40]. We suggest that muscular strength workouts when applied during the sensitive period identified (around the year of PHV) may enhance response and adaptation to training in basketballers. Further investigations combining longitudinal data with training programmes, hormonal data and kinematic information are needed.

This study is not without limitations. The first concerns the sample size and representativeness. From the original sample of 293 male adolescent basketball players, PHV was only identified in 159 out of the 160 basketballers who had complete data on 5 to 6 time points. Moreover, depending on data availability, the valid sample used to estimate peak spurts varied across muscular strength components. Although we acknowledge that this is a challenging issue, we are aware that this is a common weakness in this type of research. In the Philippaerts et al. [15] study, the original sample comprised 232 young soccer players, but from the 76 potential soccer players with complete information on 4 to 5 time points, successful fitted data was only available for 33 players. The number of players used to estimate peak spurts was never higher than 17 participants. Similar difficulties have been reported in other studies with non-athletic adolescents [2,11,13]. Second, this study used a mixed-longitudinal design with only three data waves per cohort, which may have limited the estimation of height and muscular strength spurts in some players. Nevertheless, although annual measurements would be enough to estimate age-at-PHV with high precision [41], basketball players were assessed every six-months, and the method allowed an estimation of individual velocities every three-months. Third, no data were available concerning environmental factors and/or players’ lifestyle behaviors that could affect, and at the same time, help to further explain findings. Nonetheless, such information has not been reported in previous studies. Fourth, we acknowledge that our sample is not widely representative. Although it is expected that young basketball players from the city of Porto are relatively similar to those from other Portuguese regions, generalization of the results should occur with care.

We argue that the information presented in this study may be helpful for basketball coaches to make decisions when planning strength training. It is important to clearly understand the variation in growth, maturation and development, namely across puberty, and its effects on young athletes’ responses to training programs. Since basketball is a team sport, youth team coaches must consider individual changes in growth that represent differences in performance among players. Furthermore, they should be aware that youngsters pass through periods of rapid growth that constitute great windows of opportunity to improve physical condition. If workloads are applied at the right time, adaptations to training within these sensitive periods may be optimized.

## 5. Conclusions

In conclusion, peak spurts for muscular strength components were attained coincided with the attainment of PHV and PWV or occurred within 6 months of its attainment in basketballers. This finding suggests that the timing of spurts in weight and muscular strength occurs relatively early in adolescent athletes. Additionally, it suggests that young players’ maximal gains in muscular strength are somewhat related to increases in weight and muscle mass as occurs with their non-athletic peers. Moreover, basketballers reached higher spurt intensities in most strength tests, which suggests that systematic and regular basketball training appear to boost the development of muscular strength levels in young basketballers. Our novel data enhance understanding of how strength develops in youth basketballers. Based on our findings, basketball coaches as well as strength and conditioning trainers should consider individual differences in strength development and be aware of rapid periods of growth when planning and designing muscular strength training regimes. Finally, we recommend that in the future, researchers should include information on training experience, hormonal and possible genetic markers, as well as environmental factors, to provide a more encompassing understanding of spurt timings, intensities and sequences. Moreover, researchers should investigate the effects of the application of training loads during these putative sensitive periods, as well as where a lack of adherence may have a limiting effect on athletic development.

## Figures and Tables

**Table 1:** Mean constant curves velocities for height, weight and muscular strength components aligned by months from peak height velocity (PHV).

Variables		Months from PHV (0 = PHV)												
		–18	–15	–12	–9	–6	–3	0	3	6	9	12	15	18
Height (cm·year^−1^)	mean	5.12	5.91	6.49	6.20	5.77	7.42	**7.99**	6.71	4.74	4.24	4.13	3.76	2.86
	*n*	32	40	34	50	96	99	159	130	136	110	100	88	84
Weight (kg·year^−1^)	mean	4.98	4.43	4.24	4.84	6.61	6.36	**6.89**	6.65	6.54	5.80	5.58	5.60	5.31
	*n*	30	38	31	49	85	99	144	125	127	106	95	90	82
Sit-ups (reps·year^−1^)	mean	10.19	6.72	5.65	6.93	**10.69**	8.36	8.14	6.43	6.00	4.74	5.37	4.79	8.52
	*n*	24	36	22	42	65	80	99	90	73	55	46	45	27
Handgrip (kgf·year^−1^)	mean	6.07	5.69	6.50	4.81	7.29	6.85	**8.47**	7.48	7.69	6.41	6.30	5.72	6.64
	*n*	28	39	25	46	71	99	117	110	88	84	62	59	45
SMBT (m·year^−1^)	mean	0.44	0.45	0.64	0.49	0.54	0.64	**0.75**	0.65	0.61	0.55	0.65	0.58	0.65
	*n*	17	34	21	38	65	95	114	120	98	99	69	72	56
SJ (cm·year^−1^)	mean	3.95	2.64	3.15	2.49	3.64	3.45	3.55	3.47	**3.93**	2.77	3.54	2.31	3.10
	*n*	11	21	11	28	41	60	71	80	61	62	31	40	26
CMJ (cm·year^−1^)	mean	5.66	4.28	4.07	3.82	4.88	4.46	**5.59**	4.51	5.25	3.76	3.32	3.39	3.38
	*n*	19	30	20	36	59	82	85	95	71	60	42	39	28

SMBT = seated medicine ball throw; SJ = squat jump; CMJ = countermovement jump; peak velocity values are in bold.
